# Management Strategies for Refractory Ogilvie’s Syndrome: A Case Report of Extensive Colonic Ileus in a Critically Ill 65-Year-Old Patient

**DOI:** 10.7759/cureus.77493

**Published:** 2025-01-15

**Authors:** Asim Haider, Prashant KC, Elina Shrestha, Sudiksha Regmi, Muhammad Owais, Ayesha Siddiqa

**Affiliations:** 1 Internal Medicine, Saint Vincent Medical Center, Bridgeport, USA; 2 Internal Medicine, Montefiore Medical Center, New York City, USA; 3 Cardiology, Emory University School of Medicine, Atlanta, USA; 4 Infectious Diseases, Saint Vincent Medical Center, Bridgeport, USA

**Keywords:** abdominal distension, acute colonic pseudo-obstruction, colonic obstruction, ogilvie's syndrome, refractory pseudo-obstruction

## Abstract

Ogilvie’s syndrome is characterized by acute dilatation of the colon without any anatomic obstruction. It presents with signs and symptoms of small or large bowel obstruction. The management could be challenging, especially in critically and chronically ill patients. We present a case of a 65-year-old male patient who developed extensive colonic ileus following prolonged hospitalization. We discuss different management strategies that we adopted.

## Introduction

Ogilvie’s syndrome, also known as acute colonic pseudo-obstruction (ACPO), is characterized by acute dilatation of the colon without any mechanical obstruction. It is a rare disease that is more common in elderly males over the age of 60 years [1]. It usually occurs in patients who require prolonged hospitalization or long-term care facility residents [2]. It mimics the signs and symptoms of small or large intestinal obstruction without any identifiable anatomical obstruction. An ACPO can be challenging to manage, especially in immobilized patients, and carries significant morbidity and mortality. The mortality rate varies from approximately 15% in the absence of complications to 36%-44% in patients who develop complications (for example, bowel perforation or ischemia) [3].

## Case presentation

**Figure 1:**
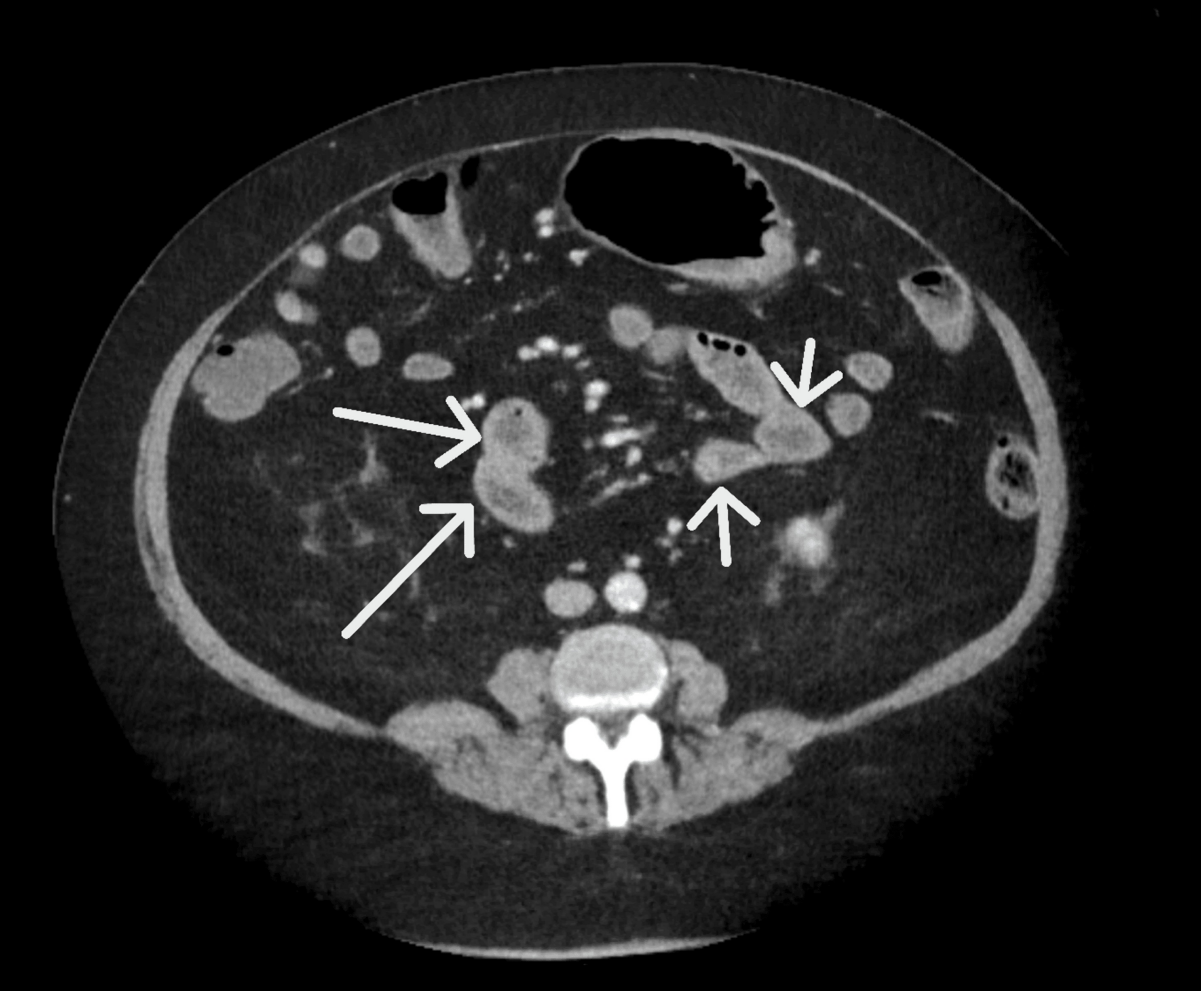
A computerized tomography scan of the abdomen showing increased thickness of small bowel loops (arrows)

A 65-year-old male patient with a medical history of hypertension, hyperlipidemia, type II diabetes mellitus, gastroesophageal reflux disease, gout, asthma, prior *Clostridium difficile* colitis, and psoriasis (on deucravacitinib) presented to the emergency department with abdominal pain for one day associated with nausea and multiple episodes of vomiting and diarrhea. He was in his usual state of health prior to this presentation. He denied any history of travel, smoking, or any other toxic habits. He denied any previous surgeries. Upon presentation, he had a blood pressure of 160/88 mmHg, a heart rate of 122 beats per minute, a respiratory rate of 22 breaths per minute, oxygen saturation of 94% on room air, and a temperature of 99°F. On physical examination, he had dry oral mucosa; the abdomen was soft, non-tender, and mildly distended with hyperactive bowel sounds. A computerized tomography (CT) scan of the abdomen and pelvis showed increased wall thickness in bowel loops, cecum, and ascending colon, suggestive of mild enterocolitis (Figure 1).

**Figure 2:**
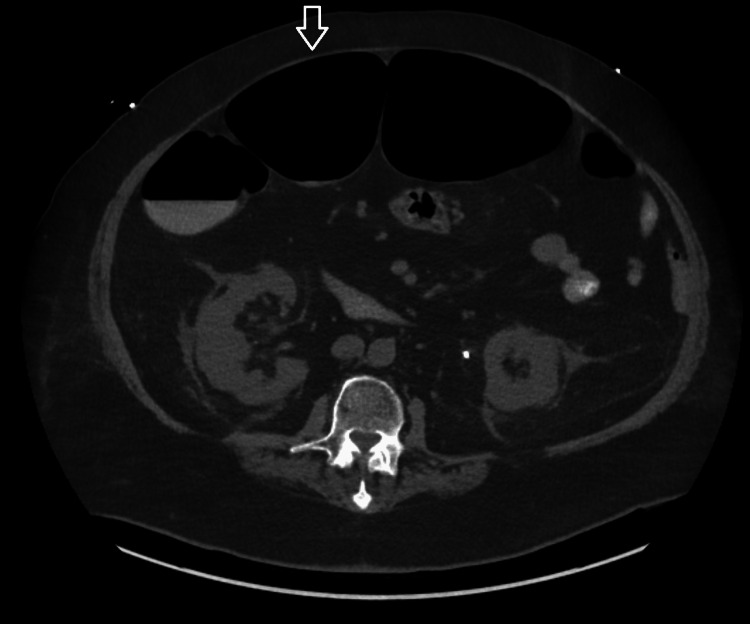
An axial computerized tomography scan of the abdomen showing colonic dilatation (arrow)

**Figure 3:**
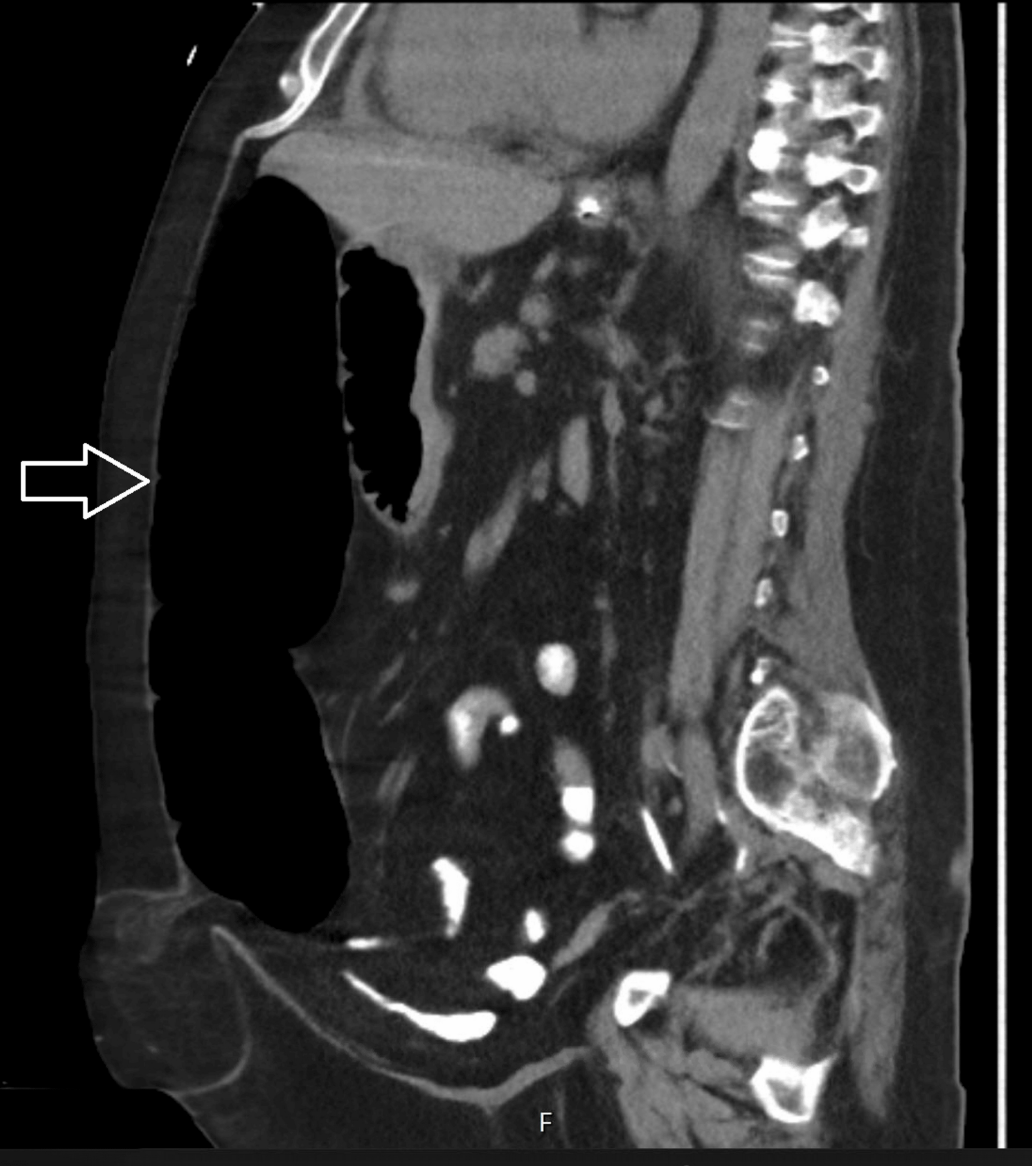
A sagittal computerized tomography scan of the abdomen showing colonic dilatation (arrow)

Stool samples tested positive for enteropathogenic *Escherichia coli* and norovirus but were negative for *Clostridium difficile*. A urinalysis was performed, which was consistent with a urinary tract infection. The patient was treated with intravenous (IV) fluids, IV ceftriaxone 1 gm daily, and appropriate electrolyte monitoring. A CT scan of the abdomen and pelvis showed an obstructing left ureteropelvic junction stone for which he underwent left ureteral stent placement. His diarrhea stopped on the third day of hospitalization, and he tolerated an oral diet. Unfortunately, his clinical course was complicated by worsening shortness of breath and acute hypoxic respiratory failure requiring intubation and mechanical ventilation. An X-ray of the chest showed opacities in the right middle and lower lung lobes concerning aspiration. Intravenous ceftriaxone was continued, and he was successfully extubated after three days of mechanical ventilation. A CT scan of the abdomen and pelvis was obtained on the 21^st^ day of hospitalization due to abdominal distention, which demonstrated marked dilation of the ascending, transverse, and descending colon with colonic diameter up to 10 cm with gradual tapering of the descending and sigmoid colon suggestive of colonic ileus. There were no features of mechanical obstruction or perforation (Figures 2, 3).

**Figure 4:**
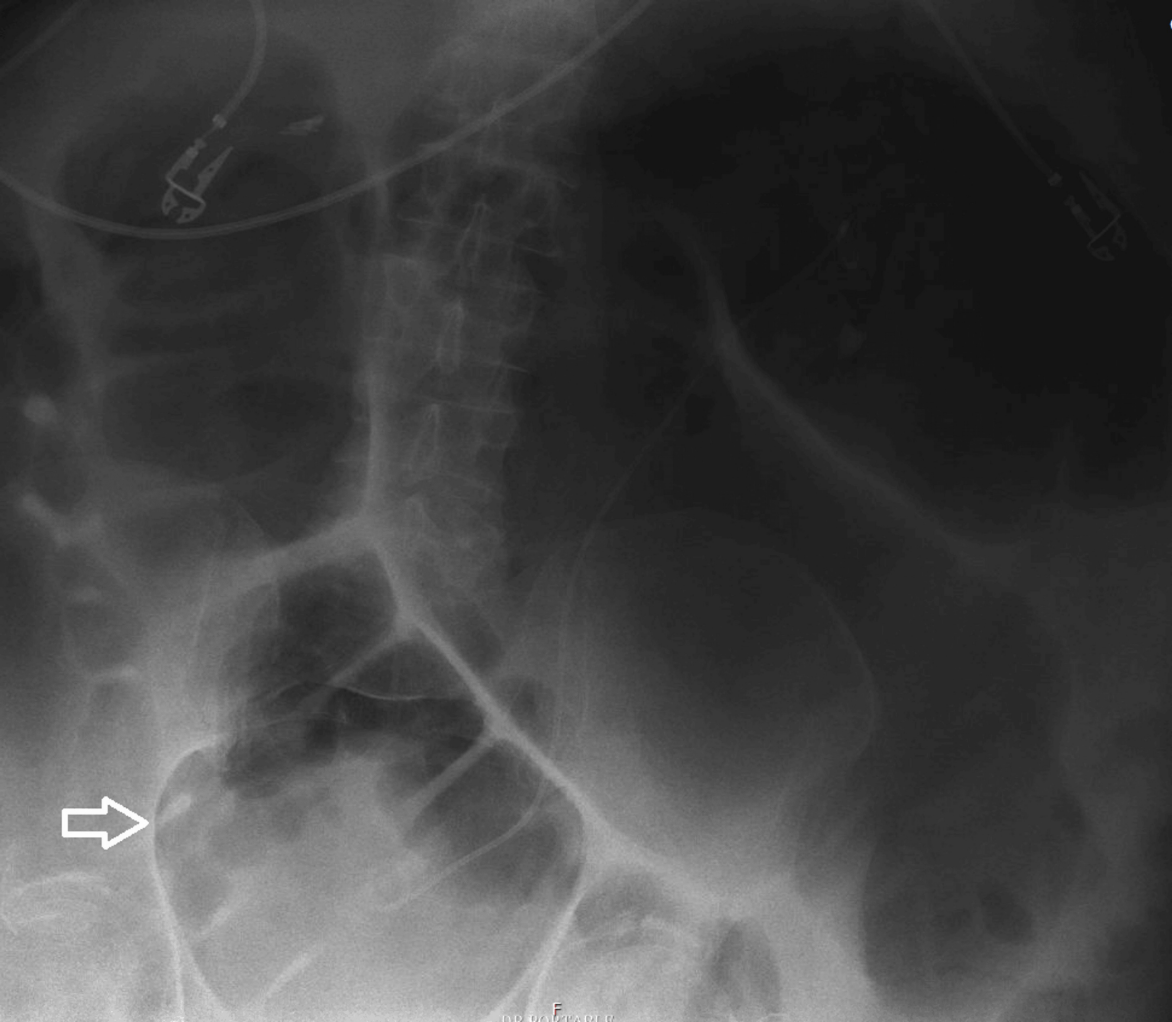
An abdominal X-ray obtained on day 26 of hospitalization showing continued colonic dilatation (arrow)

The patient was managed conservatively by maintaining nothing through the mouth and nasogastric (NG) tube decompression. Serial abdominal X-rays continue to show marked distension, including in the ascending and descending colon, with gradual tapering in the descending colon (Figure 4).

**Figure 5:**
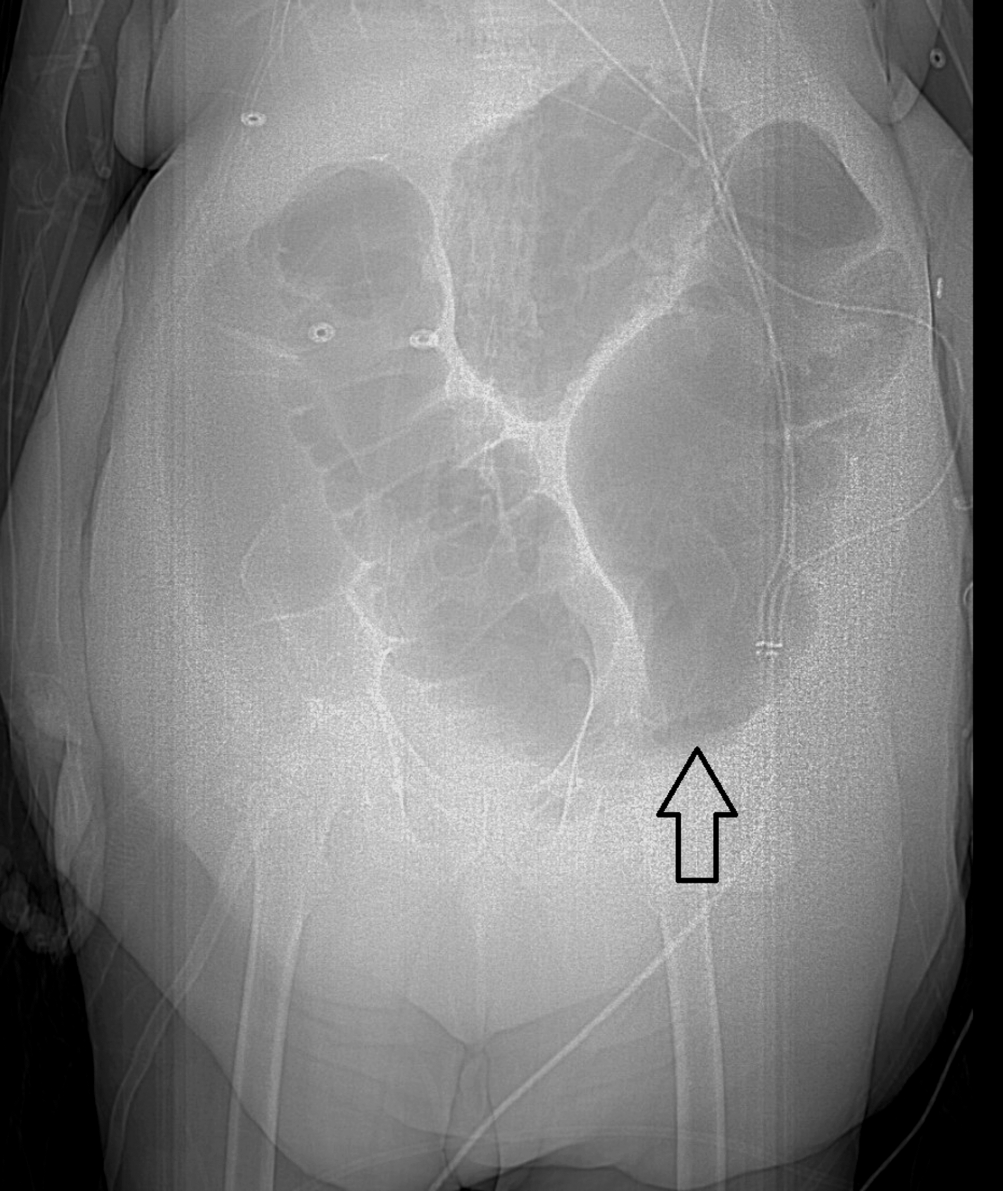
An upright abdominal X-ray showing colonic dilatation (arrow)

The patient was started on total parenteral nutrition (TPN); the NG tube was maintained, and he was started on erythromycin 250 mg orally three times daily for three days. Clinical and serial radiographic monitoring didn’t show significant improvement. Given refractory colonic dilatation, neostigmine was started. The patient received three doses of intravenous neostigmine, 2 mg daily for three days. He had a large bowel movement after the third dose, with improvement in colonic distension on the X-ray. However, over the next week, he was again noted to have abdominal distension with worsening colonic distention on X-rays (Figure 5).

**Figure 6:**
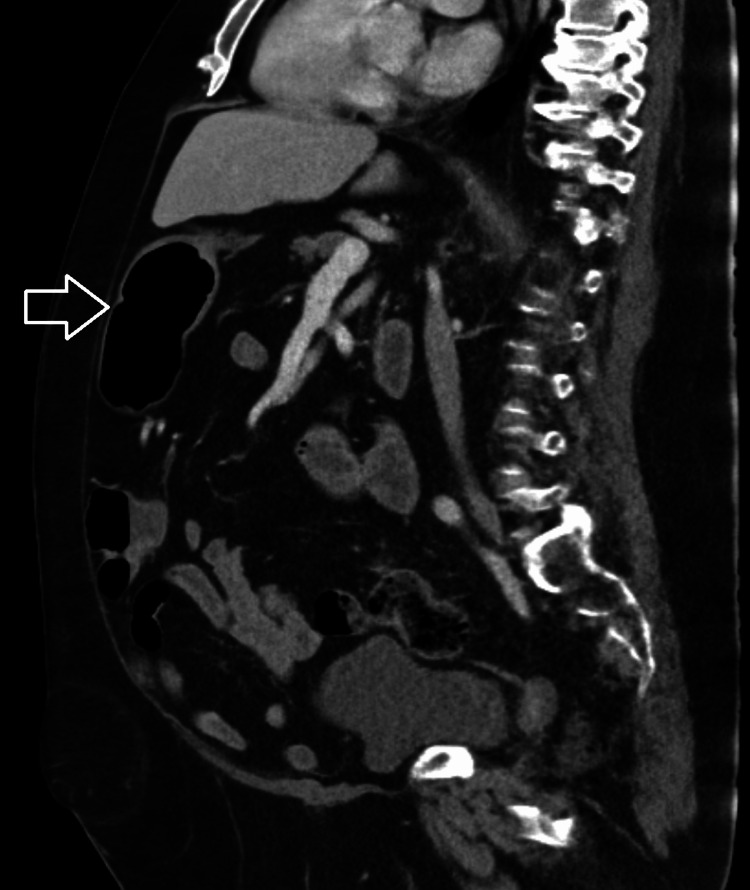
Follow-up abdominal computerized tomography scan showing improvement in colonic distention (arrow)

Gastroenterology service was consulted for colonoscopic decompression; however, given multiple comorbid conditions, he was deemed to be a poor candidate for colonoscopy. He was subsequently started on pyridostigmine 30 mg twice and azithromycin 250 mg daily. The patient showed clinical and radiological improvement after receiving therapy with pyridostigmine and azithromycin for almost one week (Figure 6).

The NG tube feeds were initiated at a slow rate. He tolerated the tube feeds well and was subsequently started on an oral diet. Total parenteral nutrition was discontinued. Azithromycin was discontinued with a plan to taper pyridostigmine on an outpatient basis. He was discharged to an acute rehabilitation facility. He is currently being followed in the outpatient setting and remains on pyridostigmine 30 mg twice daily without any recurrence of colonic symptoms. 

## Discussion

The mechanism of ACPO is not well understood. The syndrome was originally described by Sir William Ogilvie in 1948 [4]. He described it in association with retroperitoneal malignancy disrupting the autonomic nervous system to the colon. An ACPO has been described in association with neurological diseases, for example, amyotrophic lateral sclerosis [5], Parkinson's disease [6], corticobasal syndrome [7], spinal cord surgeries [8], and spinal anesthesia [9]. Antipsychotics (e.g., clozapine, olanzapine) have been associated with ACPO due to anticholinergic and antiserotonergic effects. However, the mechanism of colonic dilation in patients without autonomic dysfunction remains unclear. The other identified risk factors include trauma, severe illness, surgeries [1], electrolyte imbalance (potassium, calcium, magnesium), hypothyroidism [10], human immunodeficiency virus, and coronavirus infection.

An ACPO mostly presents with abdominal distension, pain, nausea, vomiting, and constipation. Paradoxical diarrhea has been reported in 40% of the cases [1]. Abdominal distension can also cause dyspnea. Colonic wall distension causes tension, predisposing it to ischemia and perforation. Cecal diameter of more than 10-12 centimeters and distension lasting for more than six days should be considered high risk for ischemia/perforation [11]. The diagnosis is established by a CT scan of the abdomen showing proximal colonic dilatation with a transitional point in the absence of any anatomical obstruction. The transitional point is mostly located at the splenic flexure but may extend to the rectum in some cases. The signs of systemic toxicity, for example, fever, tachycardia, altered mental status, and severe bloody diarrhea, should raise the suspicion of toxic megacolon. A plain abdominal film will show a distended colon with loss of haustra (due to the presence of submucosal edema) in such cases.

The treatment goal is to decompress the colon to minimize the risk of ischemia and perforation. A conservative approach should be pursued in the absence of signs and symptoms concerning ischemia (> 12 cm colonic dilatation, severe abdominal pain, signs of peritonitis). Conservative measures include discontinuation of medications that decrease intestinal motility (calcium channel blockers, opioids, anticholinergics), administration of intravenous fluids, electrolyte correction, decompression with a nasogastric tube, and ambulation [1]. The success rate with conservative measurements ranges from 70% to 90% [1].

Neostigmine is indicated in patients with cecal diameter > 12 cm and in cases where conservative management has failed for > 72 hours. It is an acetylcholinesterase inhibitor and requires continuous cardiac monitoring for 30 minutes post-administration. Particularly concerning side effects include bradycardia and bronchoconstriction [11]. Atropine should be available at the bedside during the administration of neostigmine. The response can be assessed via abdominal radiographs with a reduction in colonic diameter. A second dose of neostigmine can be tried after 24 hours in case of failed/partial response. Erythromycin has been tried in case reports, but the response has been inconsistent [12]. If colonic decompression is achieved successfully, oral administration of daily polyethylene glycol (PEG) is recommended to reduce the risk of recurrence [13].

Colonic decompression is recommended in patients who have failed neostigmine therapy or have contraindications to the use of neostigmine. Insufflation should be avoided or kept minimal during decompression. A colonoscopy should be performed without bowel preparation due to the risk of aspiration associated with ACPO. Opioids should be avoided for sedation as they can precipitate ACPO. A decompression tube can be placed during colonoscopy using a guide wire. This reduces the need for repeat colonoscopy for decompression and allows for the administration of PEG laxative [14]. Surgical management is recommended for patients who fail medical management or have developed complications like colonic ischemia or perforation [15].

## Conclusions

An ACPO is associated with significant morbidity and mortality, especially in men over the age of 60. Colonic ischemia and perforation are life-threatening complications. We discussed a case of resistant ACPO in a critically ill patient who failed conservative management with IV fluids, NG tube suction, and TPN. The patient required prolonged hospitalization and treatment with erythromycin, neostigmine, azithromycin, and pyridostigmine. We hope to provide clinicians with a better understanding of different management strategies that can be utilized in this rather complex syndrome that significantly prolongs hospital stays. 
